# Negative controls for bias detection within exposure-anchored self-controlled designs: applications and considerations

**DOI:** 10.1093/ije/dyag231

**Published:** 2026-10-28

**Authors:** Tu P H Trang, Marloes T Bazelier, Olaf H Klungel, Rolf H H Groenwold, Ian J Douglas, Anna Schultze, Sophie H Bots

**Affiliations:** Division of Pharmacoepidemiology and Clinical Pharmacology, Utrecht Institute for Pharmaceutical Sciences, Utrecht University, Utrecht, The Netherlands; Division of Pharmacoepidemiology and Clinical Pharmacology, Utrecht Institute for Pharmaceutical Sciences, Utrecht University, Utrecht, The Netherlands; Division of Pharmacoepidemiology and Clinical Pharmacology, Utrecht Institute for Pharmaceutical Sciences, Utrecht University, Utrecht, The Netherlands; Department of Clinical Epidemiology, Leiden University Medical Center, Leiden, The Netherlands; Department of Biomedical Data Sciences, Leiden University Medical Center, Leiden, The Netherlands; Department of Non-Communicable Disease Epidemiology, London School of Hygiene & Tropical Medicine, London, United Kingdom; Department of Non-Communicable Disease Epidemiology, London School of Hygiene & Tropical Medicine, London, United Kingdom; Division of Pharmacoepidemiology and Clinical Pharmacology, Utrecht Institute for Pharmaceutical Sciences, Utrecht University, Utrecht, The Netherlands

## Introduction

Negative control methods (NCMs), including negative control outcomes (NCOs) and negative control exposures (NCEs), are increasingly used for bias detection in epidemiological studies [1]. Taking inspiration from laboratory research, Lipsitch and colleagues [2] defined an NCO as an outcome that is not causally affected by the exposure while sharing the underlying bias structure with the primary exposure–outcome association. The definition of an NCE is identical except that it should not be causally related to the outcome. A more formal and comprehensive definition of NCMs for bias detection is provided in Supplementary Material 1, adapted from Shi and colleagues [3]. An association between an NCO and the exposure, or between an NCE and the outcome, may indicate bias in the association of interest. Frameworks have been developed for using NCMs to detect unmeasured/residual confounding [2], selection bias [4], and measurement bias [4, 5], with predominant applications in cohort designs [1].

Recently, NCMs have been applied/extended to self-controlled designs, such as the self-controlled case series (SCCS) and self-controlled risk interval (SCRI) [6, 7]. These exposure-anchored self-controlled designs are widely used in drug and vaccine safety research because of two main advantages [8, 9]. First, instead of comparing exposed and unexposed individuals, they compare exposed and unexposed time periods (focal and referent windows, respectively) within the same individual. Consequently, all confounders that stay stable over the study period are automatically controlled for regardless of whether they are measured. Second, they do not require a separate control group, which makes them more efficient to implement [10]. Illustrations of the SCCS and SCRI design are provided in Supplementary Material 2. However, they also come with their own set of considerations. First, as time-invariant confounders are automatically adjusted for, the relevant underlying bias structure should focus on time-varying factors that may differ between exposed and unexposed periods. Second, the validity of these designs rests on four assumptions, namely: (i) clearly defined outcome onset and risk period; (ii) the outcome is independently recurrent or rare; (iii) the outcome does not alter future exposure probability; and (iv) the outcome does not censor the observation period [11]. The implications of these two considerations on negative control selection have received limited attention.

In this paper, we explain the sources of bias in the SCCS/SCRI design that can be detected via NCMs, illustrate this by using directed acyclic graphs, and discuss examples of their implementation. We then discuss how the design-specific assumptions affect the selection and interpretation of NCMs. We specifically focus on NCMs for bias detection.

## Detecting bias in exposure-anchored self-controlled designs with NCMs

### Unmeasured time-varying confounding

#### Bias mechanism

Because self-controlled designs automatically adjust for time-invariant confounders, only variables that have effects or values that change between the exposed and unexposed time periods are of concern. Although adjustment methods for such time-varying confounders have been developed, these cannot address unmeasured or poorly measured confounders [10]. Examples of time-varying confounding include COVID-19 infection when studying the association between COVID-19 vaccines and myocarditis [12] and behavioural change (e.g. smoking or alcohol use) that confounds the association between antipsychotic medications and stroke [13]. Confounding by indication also represents a form of time-varying confounding, as the indication for medicine prescription is often time-dependent [14]. For instance, Hallas and colleagues demonstrated that, although penicillin has no established association with thromboembolism, it appeared harmful in an SCCS analysis because the indication for penicillin (respiratory infection) is a risk factor for thromboembolism [14].

#### Principles of selecting negative controls to detect unmeasured time-varying confounding

Let *A* and *Y* denote the exposure and outcome of interest, and *L* and *U* denote the measured and unmeasured confounders (i.e. common causes) of *A* and *Y*. In general, an ideal NCO (*N_Y_*) to detect unmeasured confounding is an outcome such that the common causes of *A* and *Y* are also the common causes of *A* and *N_Y_* (Fig. 1) [2]. Similarly, an NCE (*N_A_*) shares the same common causes with *A* and *Y*. The closer the confounding mechanisms (effect direction and magnitude) are between the primary exposure/outcome and the NCE/NCO, the more informative the negative control becomes [15]. In principle, it is sufficient for *N_Y_* and *N_A_* to share unmeasured common causes *U* with *A* and *Y*, but them sharing measured confounders *L* with *A* and *Y* could help detect residual confounding or analytic error (e.g. model misspecifications) [2]. In self-controlled designs, only time-varying factors are a relevant source of confounding, so *N_Y_* and *N_A_* do not need to share common time-invariant confounding with *A* and *Y*. Consequently, a valid NCO or NCE for an SCCS or SCRI may not be appropriate for a cohort or case–control design, and vice versa. For example, an SCCS study of COVID-19 vaccine safety used hip fractures as an NCO to evaluate unmeasured confounding arising from pandemic-related changes in healthcare utilization [16]. In contrast, if the same research question had been investigated using a cohort design comparing vaccinated individuals with index-date-matched unvaccinated individuals, then both groups would have been observed at the same stage of the pandemic, making this NCO uninformative.

**Figure:**
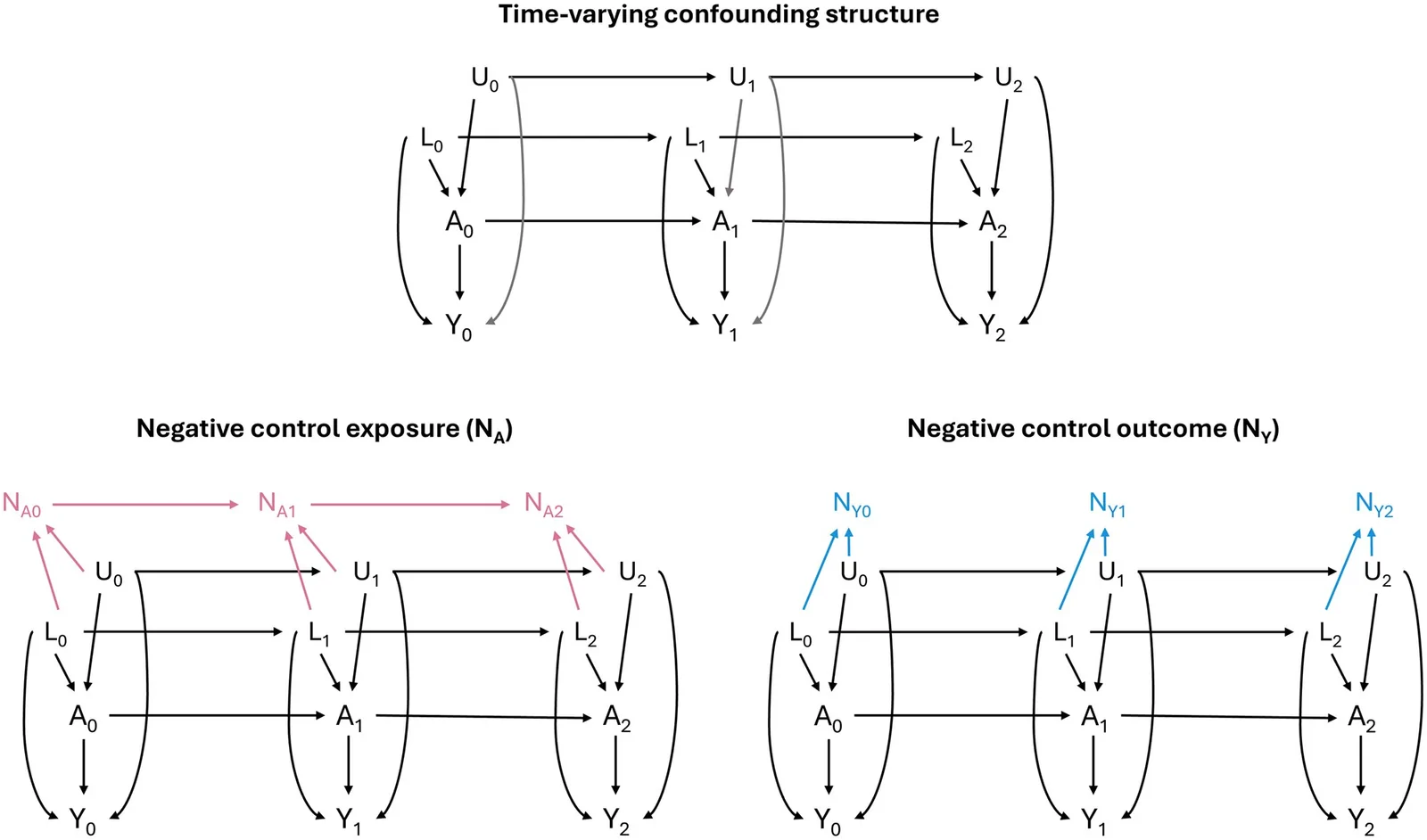
**Figure 1** Causal diagrams for time-varying confounding structure of the effect of exposure *A* on outcome *Y* in SCCS/SCRI design, along with negative control exposure (*N_A_*) and negative control outcome (*N_Y_*). *L* and *U* represent measured and unmeasured time-varying confounding, respectively. The subscripts 0, 1, and 2 represent the variables being measured at different windows during the observation period. The absence of arrows from *Y* to *Y* and *A* at a later period encodes two assumptions of the SCCS/SCRI designs: outcomes should arise independently and outcomes should not influence subsequent exposure. The absence of arrows from *A* to *Y* at a later period represents that the exposure effect is transient. Several arrows are omitted for readability: arrows from *L* and *U* to *A* and *Y* at later time points, and arrows between *L* and *U*, which implies that *L* and *U* may associate with each other and share common causes. SCCS/SCRI samples only cases, but we do not display this in the figure because it does not affect the illustrated confounding bias.

The specification of the NCE model also warrants comment. Conventionally, the $A_{E}-Y\;$model omits A, capturing confounding through both *N_A_* ← *U* → *Y* and *N_A_* ← *U* → *A* → *Y* [2]. However, when *U* affects *A* but not *Y*, the open path *N_A_* ← *U* → *A* → *Y* yields a false-positive signal despite an unconfounded *A*–*Y* relationship and adjusting for A is preferable. Because the correct model specification depends on the unknown role of *U*, we can fit both specifications and compare, treating concordance as reassuring and any discrepancy as evidence of sensitivity to this assumption.

#### Examples of negative controls applied to detect unmeasured confounding

Bots and colleagues used otitis externa (‘swimmer’s ear’) as an NCO in an SCRI study to evaluate COVID-19 vaccine safety because it shared potential unmeasured time-varying confounders with the primary outcome myocarditis (Table 1, example 1) [12]. An NCE that has similar indication to a drug exposure of interest could be used to detect confounding by indication [14]. For example, when studying fluoroquinolones and seizure risk, macrolides—which do not increase seizure risk—can serve as the NCE to detect confounding from respiratory infections, which is the indication for both drug classes (Table 1, example 2) [17].

**Table 1:** Examples of bias structures in exposure-anchored self-controlled design and the application of negative controls to detect bias.

Example	Bias structure	Exposure (*A*)	Outcome (*Y*)	Potential source of bias	Negative control exposure (*N_A_*) or negative control outcome (*N_Y_*) selection and rationale
(i) Unmeasured confounding	Figure 1, bottom-right	COVID-19 vaccine	Myocarditis	Confounding due to unmeasured time-varying factors (*U*): COVID-19 infection, other infectious origins, and influence of lockdown strategies [12]	*N_Y_*: Otitis externa There is no evidence of its association with COVID-19 vaccine and it is hypothesized to be affected by *U* in the same way as myocarditis [12]
(ii) Unmeasured confounding	Figure 1, bottom-left	Fluoroquinolones use	Seizure	Confounding by indication (*U*): respiratory infection is an indication for fluoroquinolones and is also a risk factor for seizure	*N_A_*: Macrolides Macrolides have not been shown to increase seizure risk in the literature and respiratory infection is also an indication for macrolides [17]
(iii) Selection bias + differential outcome measurement bias	Figure 2a	Influenza vaccine	Febrile seizure	Increased health-seeking behaviour (*V*) following vaccination: after receiving the vaccine, individuals can be more vigilant to monitor adverse events and seek care. Thus, the possibility of being hospitalized for the outcome (Y*) can be higher in the period immediately after vaccination than in other periods	*N_Y_*: Febrile seizure occurs between 8 and 14 days after vaccination It is outside the biologically plausible window to be caused by vaccine, while its detection (N_Y_*) is still affected by increased health-seeking behaviour [24]
(iv) Selection bias + differential outcome measurement bias	Figure 2b	COVID-19 vaccine	Pancreatitis	Limited hospital capacity during the pandemic (*U*) reduces the seeking of medical care for pancreatitis [hence the detection of pancreatitis (Y*)]. The pandemic also affects vaccination pattern [25]	*N_Y_*: Fracture There is no evidence of its association with COVID-19 vaccine. There was evidence that hospitalization for fracture (N_Y_*) was also reduced during the pandemic [26]
(v) Selection bias + differential outcome measurement bias	Figure 2a	Influenza vaccine	Asthma exacerbation	Reverse causality: outcome occurred before vaccination (*Y*) are incorrectly recorded as occurring on the day of vaccination (Y*). In the study by Tata *et al.* (2003), the general practitioners take patient history of asthma during vaccine administration and code it to that day [27]	*N_Y_*: Gout flare recorded on day of vaccination In the study setting of Tata *et al.* (2003), other medical conditions that were taken during general practitioners’ consultation but not associated with influenza vaccine, such as gout flare, could be used as negative control outcome [27].
(vi) Selection bias	Figure 2c	COVID-19 vaccine	Myocarditis	Exclude individuals with COVID-19 infection during the observation period (*S*): vaccination reduces infection risk (*A* → *S*) and infection increases myocarditis risk (*S* → *Y*). If seasonal patterns (*T*) affect both infection and myocarditis, conditioning on *S* introduces selection bias via the collider path *A* → *S* ← *T* → *Y*	*N_Y_*: Acute kidney injury Acute kidney injury is not associated with COVID-19 vaccine, while COVID-19 infection increases its risks. Acute kidney injury also exhibits seasonality similarly to myocarditis, with higher incidence in winter [28]
(vii) Differential exposure measurement error	Figure 2d	COVID-19 infection	Ischaemic stroke	The date of testing for COVID-19 (A*) is likely to be later than the date of SARS-CoV-2 infection (*A*). Hospitalization for stroke prompts testing for infection (*Y* → A*), resulting in a spike of events recorded on day of infection (Day 0) [29]	We have not found a suitable negative control for this example. A simulation study suggested to exclude day-zero events from the post-exposure focal window (risk period) to avoid this type of bias [29]

### Selection bias and exposure/outcome measurement error

#### Bias mechanism

Selection bias occurs when the analysis conditions on a common descendant of the exposure (or a cause of it) and the outcome (or a cause of it) [18]. Measurement error occurs when the analysis uses mismeasured A* and/or Y* instead of *A* and *Y*. As self-controlled designs include only detected cases and thus condition on Y*, the two biases coincide when outcome detection is associated with the exposure. In what follows, we discuss several mechanisms of selection bias and measurement error relevant to self-controlled designs, the first two of which involve both.

The first mechanism arises when awareness of exposure status (time-varying factor *V* in Fig. 2a) leads to differential outcome ascertainment/detection (Y*) between the focal and referent windows. As an example, public awareness of myocarditis after BNT162b2 vaccination likely led to the detection of mild or subclinical cases soon after vaccination, while such cases might have gone unreported had they happened before vaccination [19].

**Figure:**
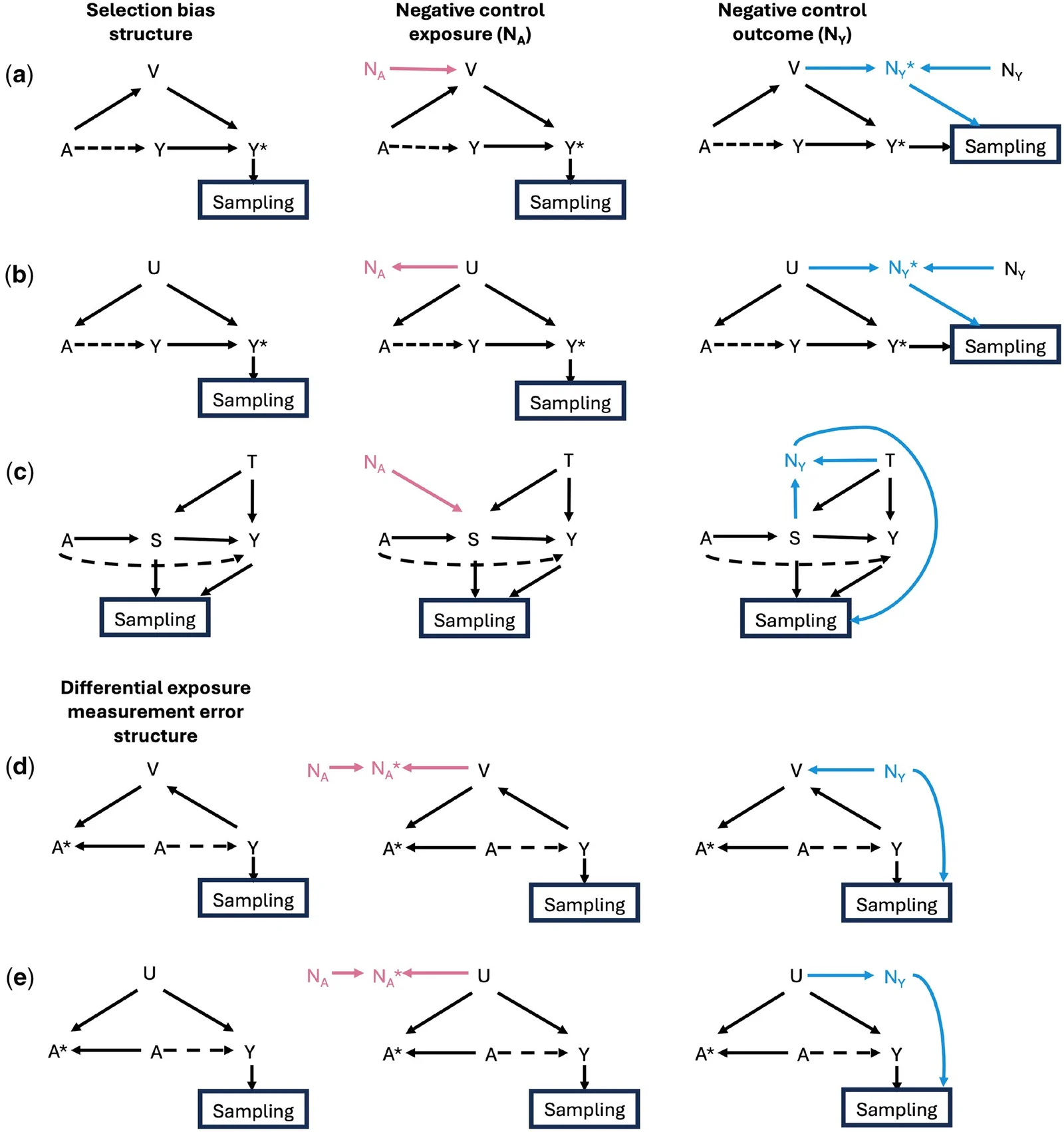
**Figure 2** Causal diagrams for common selection bias and measurement error structures of the effect of exposure *A* on outcome *Y* in exposure-anchored self-controlled design, along with the negative control exposure (*N_A_*) and negative control outcome (*N_Y_*) to detect the bias. The node ‘Sampling’ indicates that SCCS/SCRI conditions on detected cases. (a) The bias resulting from using the (mis)measured value Y* of *Y*, which is influenced by *A* via (unmeasured) factors *V*. (b) (Unmeasured) factors *U* affect both the exposure status and the outcome measurement. (a) and (b) also describe differential outcome measurement error. (c) The bias caused by selection or conditioning on a variable *S* that is a mediator between *A* and *Y*, in the presence of a mediator–outcome confounder *T*. (d, e) Differential exposure measurement error. In (d), bias arises when the occurrence of outcome *Y* influences the recording of *A* (A*) via (unmeasured) factor *V*. In (e), factor *U* affects both *Y* and the recording of *A*. Removing the arrow between *U* or *V* and *Y* results in independent, non-differential errors. When *U*, *V*, and *T* encode only time-invariant variables, these structures may lead to biased inference in the target population but valid inference in the selected population. One general principle of negative control exposure and negative control outcome to detect these biases is that they should have similar incoming or outgoing arrows to *A* and *Y*, respectively.

The second mechanism arises when the outcome ascertainment (Y*) that the analysis conditions on is affected by a time-varying factor *U* that also influences the exposure (Fig. 2b). For instance, the likelihood of diagnosis or admission may vary over time due to lockdowns or hospital capacity (Table 1, example 4) [20, 21].

The third mechanism is selection bias without measurement error, which arises if the analysis is conditioned on a variable *S* that lies on the causal pathway between the exposure *A* and outcome *Y*, in the presence of a time-varying factor *T* affecting both *S* and *Y* (Fig. 2c). For example, in studies investigating the effect of COVID-19 vaccination (*A*) on myocarditis risk (*Y*), some analyses excluded individuals having COVID-19 infection during the observation period, to control for its confounding effect [22]. However, vaccination reduces infection risk (*A* → *S*) and infection increases myocarditis risk (*S* → *Y*). If seasonal patterns (*T*) affect both infection and myocarditis, then conditioning on *S* introduces selection bias via the collider path *A* → *S* ← *T* → *Y* (Table 1, example 6).

It is important to distinguish that, in the selection bias structures described above, if the mediating or confounding factors (*V*, *U*, or *T*) are time-fixed, then self-controlled designs still yield valid inferences for the selected (i.e. included) population, but findings may not be generalizable to the (intended) target population. However, when *V*, *U*, or *T* are time-varying, then both internal validity and generalizability may be compromised.

Regarding measurement error, another concern in self-controlled designs is exposure misclassification. This may arise when prescription records are used to define drug exposure [13] or when missing vaccination status is taken as unvaccinated [10]. Differential exposure misclassification (i.e. different recording of exposure depending on the outcome status) is less likely in electronic health-records-based studies, as the recording of exposure is generally independent of the outcome. Moreover, exposed cases that are misclassified as non-exposed are less problematic in SCRI and SCCS designs that restrict to exposed cases because they do not contribute to the analysis [23]. Still, precise timing of exposure and outcome remains critical. Measurement error in either the timing and duration of exposure (Fig. 2d and e, and Table 1 example 7) or the onset of outcome (Fig. 2a and b, and Table 1 example 5) can result in the allocation of events to incorrect windows, biasing the estimates [11].

#### Principles of selecting negative controls to detect selection bias and measurement error

The general principle is that all incoming and outgoing arrows for the NCO should be similar to those of the outcome of interest, except a direct arrow from exposure *A* to the NCO (Fig. 2). Similarly, the NCE should mirror the exposure, except without a causal link to the outcome. To be most informative in detecting bias due to time-varying factors, these similarities should also match in timing. For example, to use the detection of an NCO (N_Y_*) to identify bias due to delayed detection of outcome *Y* during the pandemic (Fig. 2b), N_Y_* should be delayed to the same extent and in the same period as Y*.

In selecting negative controls to detect measurement error, attention should be paid to the similarity of the ascertainment mechanism between the primary exposure/outcome and the negative controls. To this end, Núñez *et al.* provided a helpful classification of types of outcomes and their susceptibility to outcome measurement error [5]. Selecting an NCO that is similar in clinical severity and healthcare engagement to the primary outcome increases the likelihood that they share ascertainment mechanisms. For instance, in a study of acute pancreatitis hospitalizations after COVID-19 vaccination, fractures could serve as a suitable NCO to detect the influence of changing hospital capacity on delaying outcome detection, as both are severely symptomatic conditions requiring urgent medical care.

Besides selecting a separate NCO, the primary outcome itself can serve as an NCO if measured during a period in which the exposure is unlikely to cause it. For example, to examine detection bias due to increased health-seeking behaviour after vaccination in studies of vaccine-induced febrile seizures, a period shortly after exposure but outside the biologically plausible window (e.g. 8–14 days after vaccination) could be used as a negative control focal window (Table 1, example 3) [24].

## Applicability of design-specific assumptions to negative controls in exposure-anchored self-controlled designs

The validity of exposure-anchored self-controlled designs depends on assumptions about the nature and timing of the exposure and outcome. In this section, we discuss why NCMs should also meet these design-specific assumptions alongside the general principles discussed earlier, which are summarized in Table 2. In addition, we outline how the NCE approach could be used to detect the violation of one particular assumption in the primary exposure–outcome pair. Details on the importance of the assumptions and how to check them have been discussed elsewhere [30–32].

**Table 2:** Summary of the applicability of design-specific assumptions to negative controls in exposure-anchored self-controlled designs, and methods to assess and circumvent assumption violation for negative controls.

SCCS/SCRI validity assumption	Should negative controls meet the assumption?	Methods to assess whether the negative control violates the assumption	Methods to circumvent the assumption violation
Clear-onset event	NCO to detect confounding (Fig. 1) and to detect selection bias due to conditioning on a mediator (Fig. 2c): yes, correct measurement of the timing of the event onset is needed NCO to detect selection bias due to differential outcome measurement (Fig. 2a and b): abrupt onset is not strictly required, but similar detectability to the primary outcome is advisable NCE: yes, correct measurement of the timing of the exposure is needed	Knowledge on the nature of the outcome and its delay in diagnosis or recording in the study data	The precision of the exposure, outcome and NCO/NCE measurement should match the research question: e.g. to test whether the outcome occurs within hours after exposure, the exact hour of the exposure and the outcome onset should be known [9]
Events are uncommon or arise in a non-homogenous Poisson process	NCO: yes NCE: NA	Knowledge on whether the event can be recurrent Estimation of the outcome incidence during the observation period in the target population [30] Test whether events are independently recurrent: likelihood-ratio test of the interaction between the exposure and the marginal event count; plot of the cumulative hazard of gap time between events [30]	If the occurrence of a first event is rare: restrict the analysis to first events only If events cluster in episodes but episodes can be assumed independent: restrict the analysis to the first event in each episode [30]
Events do not affect the probability of future exposure	NCO: yes, the NCO should not affect the primary exposure NCE: yes, the primary outcome should not affect the NCE	Histogram of the distribution of the interval between exposure and event times [31] Test for short-term event-dependent exposure: include the pre-exposure risk period in the standard SCCS model and use likelihood-ratio test [30]	If there can only be one exposure (e.g. single-dose vaccine): start the observation at exposure [11] Extended SCCS models incorporating counterfactual exposures [30] If event-dependent exposure is short-lived: include the pre-exposure risk period in the standard SCCS model (i.e. exclude that period from the referent window) [31]
Events do not censor or affect the observation period	NCO: yes NCE: NA	Histogram of the interval between event time and censoring Test whether censoring biases the effect estimates: fit SCCS model with interaction term between exposure and censoring indicator, and compare likelihood with model without interaction [31]	Extended SCCS models for rare, nonrecurrent interferent events [33]

### Assumption 1: clearly defined outcome onset and risk period

The SCCS/SCRI methods compare outcome incidence in focal versus reference windows, thus it requires event onset to be accurately measured to assign events to the correct window [34]. These designs are therefore ill-suited for outcomes with insidious onset or diagnostic delays. Correct measurement of exposure timing is equally important to define the focal/control window.

For NCOs, the relevance of this assumption depends on the bias being tested. If the NCO reflects the effect of the mediating, confounding, or selecting factors (*U*, *V*, *S*, *T* in Figs 2–4) on ‘experiencing’ the outcome of interest (Figs 2 and 4c–e), then the NCO timing must be measured accurately to reflect effects during relevant windows. In an extreme example, suppose COVID-19 infection is a confounder of a vaccine–adverse event association, and osteoarthritis is used as an NCO due to a presumed association between COVID-19 infection and osteoarthritis. Because osteoarthritis has a long latency, cases occurring during the observation period are unlikely to be caused by recent COVID-19 infection. The negative control analyses would then not inform us about the likelihood of unmeasured confounding due to COVID-19.

On the other hand, if we are using the detection of the NCO (N_Y_*) to test the factors that affect the ‘detection’ of the outcome of interest (Y*) (Fig. 2a and b), then the variable of interest is the detected NCO (i.e. N_Y_*), not the true onset of *N_Y_*. However, the ‘detectability’ (such as the severity and acuteness) of the NCO should still be similar to that of the primary outcome, so that they are more likely to share mechanisms affecting their detection. In the above example of the influence of changing hospital capacity on delaying pancreatitis detection, if hypertension (a condition with unclear onset) is chosen as the NCO, it may reflect a different degree of delaying detection than pancreatitis.

### Assumption 2: events are uncommon or arise in a non-homogenous Poisson process

The conditional Poisson models underpinning the SCCS/SCRI assume that events are either independently recurrent or rare and nonrecurrent. If this assumption is violated, then time-invariant confounders may not be fully controlled for [30]. This applies to both the outcome of interest and the NCO—it is therefore important to ensure that any NCO meets this assumption, to ensure valid inference.

Literature has suggested potential approaches to address this limitation that depend on the nature of the recurrence. If events cluster in episodes but episodes can be assumed to be independent (e.g. migraine attacks), then the standard methods can be applied to the first event in each episode. Alternatively, if recurrent events are not independent but the occurrence of a first event is rare (e.g. myocardial infarction), then restricting the SCCS analysis to first events only can preserve model validity [34].

### Assumption 3: events do not affect the probability of future exposure

SCCS/SCRI designs assume that events do not influence subsequent exposure. This consideration applies to the primary exposure and outcome, as well as NCOs and NCEs. For example, if influenza is used as an NCO in a COVID-19 vaccine study, symptomatic individuals may defer vaccination, leading to underrepresentation of influenza events in the pre-exposure window and upward bias. Conversely, if events increase the likelihood of exposure (e.g. road accidents leading to nonsteroidal anti-inflammatory drug (NSAID) use), it induces a downward bias [30].

Strategies that mitigate the assumption violation for the primary exposure/outcome can also be applied to the NCO/NCE. First, if there is only one exposure and observation starts at exposure, the analysis is free of this assumption [11]. Second, extended SCCS models incorporating counterfactual exposures can explicitly account for event-dependent exposure [30]. Another approach is to exclude a short pre-exposure period from the referent window, but simulations suggest this may overcorrect bias [31].

On a related note, to check this assumption for the primary exposure–outcome pair, we propose that the NCE method could be applied in addition to the standard methods of plotting the distribution of the interval between exposure and event times or estimating the relative incidence in a pre-exposure period [31]. Specifically, if an active comparator exists that does not cause the outcome but is expected to be affected by a prior outcome similar to the primary exposure, then estimating the association between the NCE and the primary outcome could give an indication on the existence of bias due to violation of the event-independent exposure assumption. Unlike standard checks, this approach may help distinguish between violations that induce meaningful bias and those with negligible impact, where correction could paradoxically introduce new bias [31].

### Assumption 4: events do not censor or affect the observation period

When selecting an NCO, the assumption that events do not affect the observation period should be met. This assumption ensures that conditioning on the observation period does not alter the incidence function and bias the estimate. The assumption may fail if the event increases short-time mortality (e.g. stroke). Violation of this assumption can be explored by plotting event-to-censoring intervals and be addressed by using extended SCCS models for rare, nonrecurrent interferent events [33].

## Further considerations

Several caveats of NCMs for bias detection exist across all study designs. First, negative controls may lack specificity with respect to the type(s) of bias they are meant to detect [15]. If, for example, both unmeasured confounding and selection bias influence the NCO, we cannot differentiate between them. Second, NCMs may also lack sensitivity to provide a definitive test for absence of bias. A null result could occur due to different reasons: different biases with opposite directions cancelling one another out, low statistical power, or insufficient similarity of the bias structure between the NCE/NCO and the primary exposure/outcome [15]. It is thus important to ensure that the NCE/NCO has adequate power to detect the suspected bias [3]. Third, the magnitude of bias cannot be inferred from the NCM results without extra assumptions [2]. Interpretation of NCM results can also be compromised if the negative controls are measured with error [35]. Appropriate negative control selection requires domain expertise and a clear understanding of likely bias sources in the study context.

## Conclusion

The use of negative controls to assess validity in exposure-anchored self-controlled designs has received growing attention. In this paper, we illustrated how time-varying confounding, selection bias, and measurement error could arise in these designs and how NCMs could be used to detect these biases. We highlighted that the utility of NCMs relies heavily on the fulfilment of both the assumptions regarding the bias structure and the specific assumptions of the exposure-anchored self-controlled designs. Thorough consideration should be taken in selecting negative controls and their results should be interpreted carefully in light of the limitations and assumptions of the NCMs and the study designs that are used.

## Ethics approval

Not applicable, as no data were used.

## Supplementary Material

dyag231_Supplementary_Data

## Data Availability

No new data were generated or analysed in support of this research.

## References

[1] Zafari Z , Park JE, Shah CH et al The state of use and utility of negative controls in pharmacoepidemiologic studies. Am J Epidemiol 2024;193:426–53. 10.1093/aje/kwad201 PMC11484649 37851862

[2] Lipsitch M , Tchetgen Tchetgen E, Cohen T. Negative controls: a tool for detecting confounding and bias in observational studies. Epidemiology (Fairfax) 2010;21:383–8. 10.1097/EDE.0b013e3181d61eeb PMC3053408 20335814

[3] Shi X , Miao W, Tchetgen ET. A selective review of negative control methods in epidemiology. Curr Epidemiol Rep 2020;7:190–202. 10.1007/s40471-020-00243-4 PMC8118596 33996381

[4] Arnold BF , Ercumen A, Benjamin-Chung J et al Brief report: negative controls to detect selection bias and measurement bias in epidemiologic studies. Epidemiology (Fairfax) 2016;27:637–41. 10.1097/ede.0000000000000504 PMC4969055 27182642

[5] Isaac N , Anthony AM. Detection bias and the role of negative control outcomes. BMJ Med 2025;4:e001336. 10.1136/bmjmed-2025-001336 PMC12172073 40529208

[6] Rudan I , Katikireddi SV, Kerr S et al Selecting the most informative positive and negative controls for self-controlled case series (SCCS): rationale, approach, and lessons from studies investigating the safety of COVID-19 vaccines. J Glob Health 2024;14:03037. 10.7189/jogh.14.03037 PMC11301617 39105317

[7] Schuemie MJ , Arshad F, Pratt N et al Vaccine safety surveillance using routinely collected healthcare data—an empirical evaluation of epidemiological designs. Front Pharmacol 2022;13:893484. 10.3389/fphar.2022.893484 PMC9299244 35873596

[8] Gault N , Castañeda-Sanabria J, De Rycke Y et al Self-controlled designs in pharmacoepidemiology involving electronic healthcare databases: a systematic review. BMC Med Res Methodol 2017;17:25. 10.1186/s12874-016-0278-0 PMC5299667 28178924

[9] Bots SH , Brown J, Wong AYS et al Core concepts: self-controlled designs in pharmacoepidemiology. Pharmacoepidemiol Drug Saf 2025;34:e70071. 10.1002/pds.70071 PMC11729261 39805806

[10] Lai LY , Arshad F, Areia C et al Current approaches to vaccine safety using observational data: a rationale for the EUMAEUS (Evaluating Use of Methods for Adverse Events Under Surveillance-for Vaccines) study design. Front Pharmacol 2022;13:837632. 10.3389/fphar.2022.837632 PMC8980923 35392566

[11] Whitaker HJ , Hocine MN, Farrington CP. The methodology of self-controlled case series studies. Stat Methods Med Res 2009;18:7–26. 10.1177/0962280208092342 18562396

[12] Bots SH , Belitser S, Groenwold RHH et al Applying two approaches to detect unmeasured confounding due to time-varying variables in a self-controlled risk interval design evaluating COVID-19 vaccine safety signals, using myocarditis as a case example. Am J Epidemiol 2025;194:208–19. 10.1093/aje/kwae172 PMC11735966 38960670

[13] Douglas IJ , Smeeth L. Exposure to antipsychotics and risk of stroke: self controlled case series study. BMJ. 2008; 337: a1227. 10.1136/bmj.a1227 PMC2526549 18755769

[14] Hallas J , Whitaker H, Delaney JA et al The use of active comparators in self-controlled designs. Am J Epidemiol 2021;190:2181–7. 10.1093/aje/kwab110 33861309

[15] Penning de Vries BB , Groenwold RH. Negative controls: concepts and caveats. Stat Methods Med Res 2023;32:1576–87. 10.1177/09622802231181230 PMC10515451 37338976

[16] Torabi F , Bedston S, Lowthian E et al Risk of thrombocytopenic, haemorrhagic and thromboembolic disorders following COVID-19 vaccination and positive test: a self-controlled case series analysis in Wales. Sci Rep 2022;12:16406. 10.1038/s41598-022-20118-6 PMC9524320 36180455

[17] Wanleenuwat P , Suntharampillai N, Iwanowski P. Antibiotic-induced epileptic seizures: mechanisms of action and clinical considerations. Seizure 2020;81:167–74. 10.1016/j.seizure.2020.08.012 32827980

[18] Hernan MA , Robins JM. Causal Inference: What If. Chapman & Hall/CRC, 2020.

[19] Patone M , Mei XW, Handunnetthi L et al Risks of myocarditis, pericarditis, and cardiac arrhythmias associated with COVID-19 vaccination or SARS-CoV-2 infection. Nat Med 2022;28:410–22. 10.1038/s41591-021-01630-0 PMC8863574 34907393

[20] Mackinnon ES , Anderson T, Raggi P et al Trends in major adverse cardiac events and healthcare resource utilization during the COVID-19 pandemic in Alberta, Canada. CJC Open 2023;5:719–27. 10.1016/j.cjco.2023.06.004 PMC10286559 37366514

[21] Pommier T , Benzenine E, Bernard C et al Trends of myocarditis and endocarditis cases before, during, and after the first complete COVID-19-related lockdown in 2020 in France. Biomedicines 2022;10:1231. 10.3390/biomedicines10061231 PMC9219624 35740252

[22] Fan M , Lai FTT, Cheng FWT et al Risk of carditis after three doses of vaccination with mRNA (BNT162b2) or inactivated (CoronaVac) covid-19 vaccination: a self-controlled cases series and a case-control study. Lancet Reg Health West Pac 2023;35:100745. 10.1016/j.lanwpc.2023.100745 PMC10039395 37360861

[23] Baker MA , Lieu TA, Li L et al A vaccine study design selection framework for the postlicensure rapid immunization safety monitoring program. Am J Epidemiol 2015;181:608–18. 10.1093/aje/kwu322 25769306

[24] Rowhani-Rahbar A , Klein NP, Dekker CL et al Biologically plausible and evidence-based risk intervals in immunization safety research. Vaccine 2012;31:271–7. 10.1016/j.vaccine.2012.07.024 22835735

[25] Du Z-C, Zhang J, Li X-J et al Impact of COVID-19 pandemic on acute pancreatitis presentations, management, and in-hospital outcomes: a single-center, retrospective observational study from the northeast of China. Therap Adv Gastroenterol 2022;15:17562848221145552. 10.1177/17562848221145552 PMC9806203 36600685

[26] Lv H, Zhang X, Wang J et al Short-term effects of COVID-19 on the risk of traumatic fractures in China cities. Sci Rep 2022;12:6528. 10.1038/s41598-022-10531-2 PMC9020760 35444225

[27] Tata LJ, West J, Harrison T et al Does influenza vaccination increase consultations, corticosteroid prescriptions, or exacerbations in subjects with asthma or chronic obstructive pulmonary disease? Thorax 2003;58:835–9. 10.1136/thorax.58.10.835 PMC1746473 14514932

[28] Selby NM. Acute kidney injury change s with the seasons. Nephrol Dial Transplant 2018;33:1281–3. 10.1093/ndt/gfy070 29635645

[29] Fonseca-Rodríguez O, Fors Connolly AM, Katsoularis I, Lindmark K, Farrington P. Avoiding bias in self-controlled case series studies of coronavirus disease 2019. Stat Med 2021;40:6197–208. 10.1002/sim.9179 PMC8661887 34470078

[30] Farrington P , Whitaker H, Weldeselassie YG. Self-Controlled Case Series Studies: A Modelling Guide with R. 1st ed. New York: Chapman and Hall/CRC, 2018.

[31] Whitaker HJ , Ghebremichael-Weldeselassie Y, Douglas IJ et al Investigating the assumptions of the self-controlled case series method. Stat Med 2018;37:643–58. 10.1002/sim.7536 29094391

[32] Whitaker HJ , Ghebremichael-Weldeselassie Y. Self-controlled case series methodology. Annu Rev Stat Appl 2019;6:241–61. 10.1146/annurev-statistics-030718-105108

[33] Farrington CP , Whitaker HJ, Hocine MN. Case series analysis for censored, perturbed, or curtailed post-event exposures. Biostatistics 2009;10:3–16. 10.1093/biostatistics/kxn013 18499654

[34] Whitaker HJ , Paddy Farrington C, Spiessens B et al Tutorial in biostatistics: the self-controlled case series method. Stat Med 2006;25:1768–97. 10.1002/sim.2302 16220518

[35] Sanderson E , Macdonald-Wallis C, Davey Smith G. Negative control exposure studies in the presence of measurement error: implications for attempted effect estimate calibration. Int J Epidemiol 2018;47:587–96. 10.1093/ije/dyx213 PMC5913619 29088358

