# Supplementary material for: Negative controls for bias detection within exposure-anchored self-controlled designs: applications and considerations
Source: Int J Epidemiol. 2026 Oct 28;55(6):dyag231. doi: 10.1093/ije/dyag231 (PMC13727053; doi:10.1093/ije/dyag231)
Supplement: Supplementary file 1 [file dyag231_supplementary_data.zip › ije-2025-09-1837-File005.docx]

**Supplementary Material 1: Definitions, assumptions and an example of negative controls in the setting of a point exposure and outcome**

In this section, we provide the definitions, assumptions and their mathematical representation of negative controls for bias detection, under the potential outcome (counterfactual) framework and the setting of a point exposure and outcome. A more detailed description of the assumptions is provided by Shi et al. (1).

Consider an observational study that aims to assess the causal effect of a binary exposure $A = \{0, 1\}$ on an outcome Y. Under the potential outcome framework, we assume that for each subject in the target population, there exist a pair of potential outcomes $(Y(1), Y(1))$, corresponding to their potential outcome under exposure $A = 0$ and $A = 1$. In the observational study, we only observed one version of the outcome Y corresponding to their observed exposure $A$. Denote $L$ the measured confounders and $U$ the unmeasured confounders of the association between $A$and $Y$. $A$and $Y$ are sometimes referred to as the primary exposure and primary outcome.

To infer causal effect of $A$ on $Y$, the assumptions needed are:

**Assumption 1 (Consistency):** $Y(a) = Y$when $A = a$. That is, the exposure is clearly defined such that an individual that received exposure $A$equal to $a$ has observed outcome $Y$ equal to their potential outcome $Y(a)$

**Assumption 2 (latent ignorability):** $A \perp Y\left( a \right) | L, U$. That is, the exposure and the outcome of interest are independent given the measured and unmeasured confounders.

Suppose that in the study, one also observed a second outcome $N_{Y}$ and/or a second exposure $N_{A}$. Denote $Y\left( a, na \right)$and $N_{Y}(a, na)$ the corresponding counterfactual values of the primary and second outcome had the primary and second exposure been set to $(a, na)$.

$N_{Y}$ and $N_{A}$ are formally defined as negative control outcome and negative control exposure, respectively, if the following assumptions hold:

**Assumption 3 (negative control outcome):**

3.1. $N_{Y}\left( a, na \right)=N_{Y} L,U$ and

3.2. $N_{Y} \perp A | L,U$.

**Assumption 4 (negative control exposure):**

4.1. $Y\left( a, na \right)=Y\left( a \right)| L,U$ and

4.2. $N_{A} \perp(Y\left( a \right), N_{Y}) | L,U$.

Assumption 3 and 4 indicate the following: 1) there is no remaining unmeasured common cause between $(A, N_{A})$ and $(Y, N_{Y})$ conditional on $(L, U)$ (assumption 3.2 and 4.2); 2) there is no causal effect of $N_{A}$ on $Y$ conditional on $U, A$, and $L$ (assumption 4.1), and there is no causal effect of $A$ and $N_{A}$ on $N_{Y}$ conditional on $U$ and $L$ (assumption 3.1).

To use the negative control exposure $N_{A}$ and/or the negative control outcome $N_{Y}$ to detect bias (e.g. bias due to unmeasured confounding U), the following assumption is required:

**Assumption 5 (U-comparability):** $N_{Y}∦U | C$ and $N_{A}∦U|C, E$.

The U-comparability assumption states that unmeasured confounder $U$ of $A-Y$ association are identical to those of the $A-N_{Y}$ association and $N_{E}-Y$ association, such that a non-null $A-N_{Y}$ or $N_{E}-Y$ association can be attributed to $U$.

Figure S1 illustrates a directed acyclic graph that encodes the above assumptions and an applied example of negative control outcome (NCO) and negative control exposure (NCE). In a cohort study of the effect of participation in mammography-screening program (A) on death from breast cancer (Y), there were concerns over residual bias from healthiness and health-seeking behaviors (U) (2). Dental-care participation ($N_{A}$) is an NCE that satisfies assumption 4, as it is likely to reflect healthiness and health-seeking behaviors, while it is unlikely to cause breast-cancer mortality. Likewise, death from causes other than breast cancer ($N_{E})$ could be an NCO satisfying assumption 3 because it could be influenced by healthiness and health-seeking behaviors, while not being related to mammography screening. The relationships between measured confounders (L) and the NCE and NCO are not strictly required for negative control for bias detection and thus not included in the figure.

Figure S1. Directed acyclic graph for causal associations between variables in a negative control analysis. A represents the exposure of interest; Y, outcome of interest; U, unmeasured confounders; N_A_, negative control exposure; N_Y_, negative control outcome

Reference:

1.	Shi X, Miao W, Tchetgen ET. A Selective Review of Negative Control Methods in Epidemiology. Curr Epidemiol Rep. 2020;7(4):190-202.

2.	Lousdal ML, Lash TL, Flanders WD, Brookhart MA, Kristiansen IS, Kalager M, et al. Negative controls to detect uncontrolled confounding in observational studies of mammographic screening comparing participants and non-participants. Int J Epidemiol. 2020;49(3):1032-42.
