# Supplementary material for: Negative controls for bias detection within exposure-anchored self-controlled designs: applications and considerations
Source: Int J Epidemiol. 2026 Oct 28;55(6):dyag231. doi: 10.1093/ije/dyag231 (PMC13727053; doi:10.1093/ije/dyag231)
Supplement: Supplementary file 1 [file dyag231_supplementary_data.zip › ije-2025-09-1837-File006.docx]

**Supplementary Material 2: Key features and examples of self-controlled case series and self-controlled risk interval design**

**Self-controlled case series design**

The self-controlled case series (SCCS) design aims to answer an underlying research question concerning the temporal relationship between exposure and outcome: given that an exposure occurs at a particular time, what is the probability that the outcome occurs shortly thereafter? [1]

In the SCCS design, only individuals who experience the outcome of interest (cases) are included in the analysis. These individuals are followed over a defined **observation period**, during which both the exposure and the outcome may occur. Within this observation period, the SCCS design specifies **focal (risk) windows** and **referent (control) windows** relative to the timing of exposure [2]. The focal window represents the period during which the risk of the outcome is hypothesized to be elevated following exposure. In contrast, the referent window represents periods during which the risk of the outcome reflects the baseline incidence. Typically, all time within the observation period outside the focal window is considered part of the referent window. Importantly, the observation period does not terminate at the time the outcome occurs.

For each individual, the incidence of the outcome during the focal window is compared with the incidence during the referent window. Differences in person-time contributed by these windows are accounted for by including the duration of each window as an **offset** in the statistical model. The analysis is commonly conducted using **conditional Poisson regression** to estimate **incidence rate ratios**. Because comparisons are made within individuals, all **time-invariant confounders** are inherently controlled.

An example of the SCCS design is the study of Walker et al (2022) investigating the risk of neurological events, including Bell’s palsy following the first COVID-19 vaccination, using data in the OpenSAFELY platform (Figure S2) [3]. Eligible participants were adults who had been continuously registered with a general practice for at least one year prior to 1 July 2020. The study period, which also defined each individual’s observation period, extended from 1 July 2020 to 7 July 2021. The SCCS analysis included individuals who both received a COVID-19 vaccination and experienced a new episode of Bell’s palsy during the observation period. Following each vaccination, a focal window of 4–28 days was defined. The first four days after vaccination were treated as a transition (induction) window, during which the occurrence of the outcome was considered biologically implausible. Additionally, the 28 days preceding vaccination were treated as a pre-exposure transition window. All remaining time within the observation period, outside the focal and transition windows, constituted the referent window. The incidence rate ratio comparing the focal and referent windows was estimated using conditional Poisson regression.

Figure S2. Illustration of the time-windows for the example of a self-controlled case series and a self-controlled risk interval design to answer the research question whether COVID-19 vaccines increase the risk of Bell’s Palsy.

**Self-controlled risk interval design**

The self-controlled risk interval (SCRI design) is a variant of the SCCS design. The principal distinction from the SCCS design is that the SCRI design defines one or more discrete referent windows of fixed duration, and disregard the remaining follow-up time during the study period. Referent windows can be positioned before or after the exposure, or both. Compared with the SCCS design, the SCRI design is generally simpler to implement in its basic form, and the shorter follow-up period allows analyses to be conducted more quickly [2].

The example above of the association between COVID-19 vaccines and Bells’s Palsy can be investigated with an SCRI design (Figure S2). In this hypothetical study, vaccinated individuals who developed Bell’s Palsy between 1 July 2020 and 7 July 2021 are eligible for inclusion. The period of days 4 to 28 after vaccination can be chosen as the focal window, and the periods of days −90 to −30 before vaccination can be used as the referent window. The period immediately preceding vaccination (days −29 to −1) and immediately after vaccination (days 0 to 3) are treated as a transition window. The observed time of each individual outside these windows are discarded. Conditional Poisson regression can be applied to estimate the incidence rate ratio comparing the focal and referent windows.

Reference:

1.	Whitaker HJ, Paddy Farrington C, Spiessens B, Musonda P. Tutorial in biostatistics: the self-controlled case series method. *Statistics in Medicine*. 2006;25(10):1768-1797. doi:<https://doi.org/10.1002/sim.2302>

2.	Bots SH, Brown J, Wong AYS, et al. Core Concepts: Self-Controlled Designs in Pharmacoepidemiology. *Pharmacoepidemiol Drug Saf*. Jan 2025;34(1):e70071. doi:10.1002/pds.70071

3.	Walker JL, Schultze A, Tazare J, et al. Safety of COVID-19 vaccination and acute neurological events: A self-controlled case series in England using the OpenSAFELY platform. *Vaccine*. Jul 30 2022;40(32):4479-4487. doi:10.1016/j.vaccine.2022.06.010
